# Supplementary material for: Coupling carbon and energy fluxes in the North Pacific Subtropical Gyre
Source: Nat Commun. 2019 Apr 26;10:1895. doi: 10.1038/s41467-019-09772-z (PMC6486601; doi:10.1038/s41467-019-09772-z)
Supplement: Supplementary file 1 — Supplementary Information [file 41467_2019_9772_MOESM1_ESM.pdf]

Coupling carbon and energy fluxes in the North Pacific Subtropical Gyre

Grabowski et al.

Supplementary Information

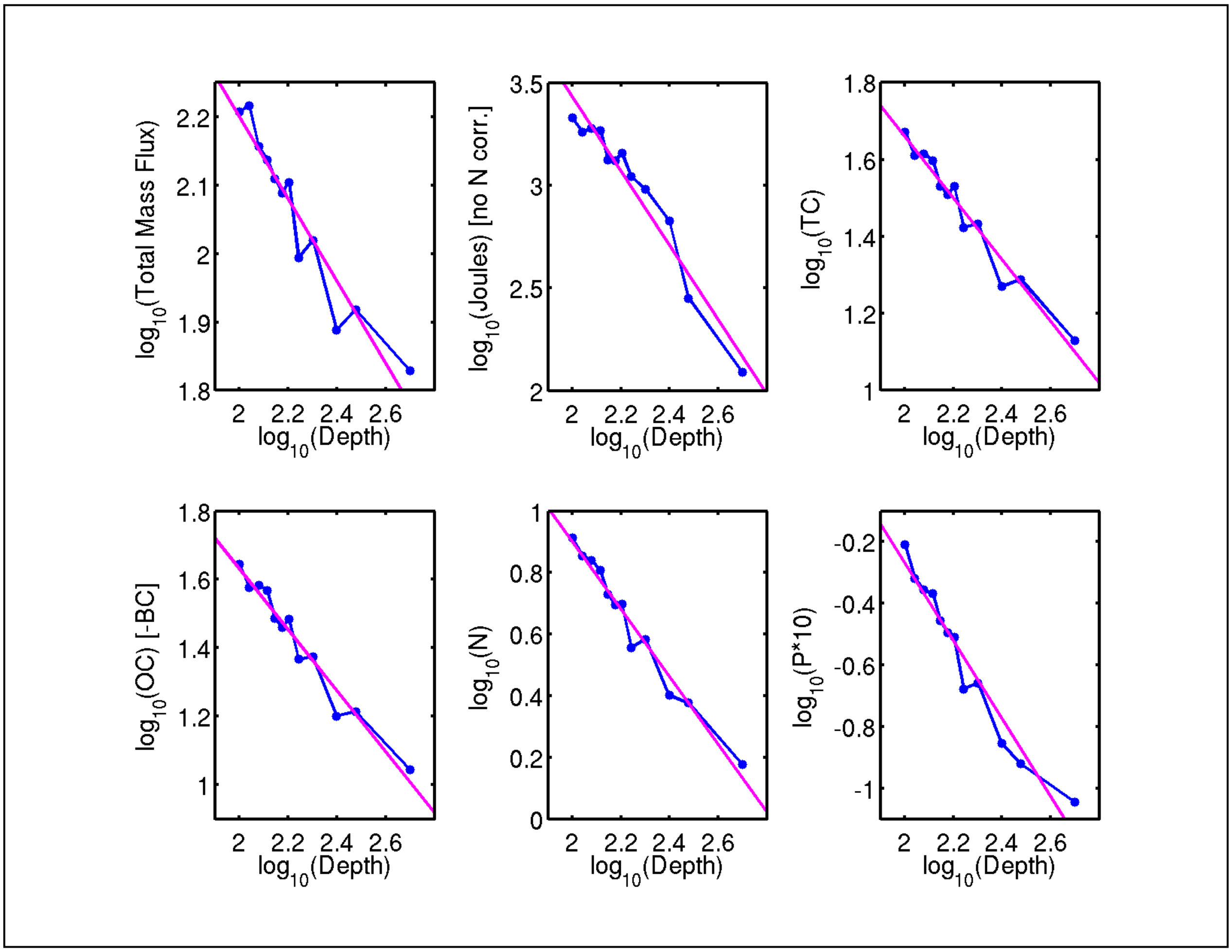

Supplementary Figure 1. Mass, element and energy flux profiles. Plots of $\log_{10}$ mass, elemental (OC, TC, N and P) and energy fluxes versus $\log_{10}$ depth (m) for the particulate matter collected in the free-drifting sediment trap experiment. The magenta lines are the linear regression fits that were used to estimate the flux at 100 m and the flux attenuation co-efficient (b) reported in Figs. 1 and 2 and Supplementary Data Fig. 2.

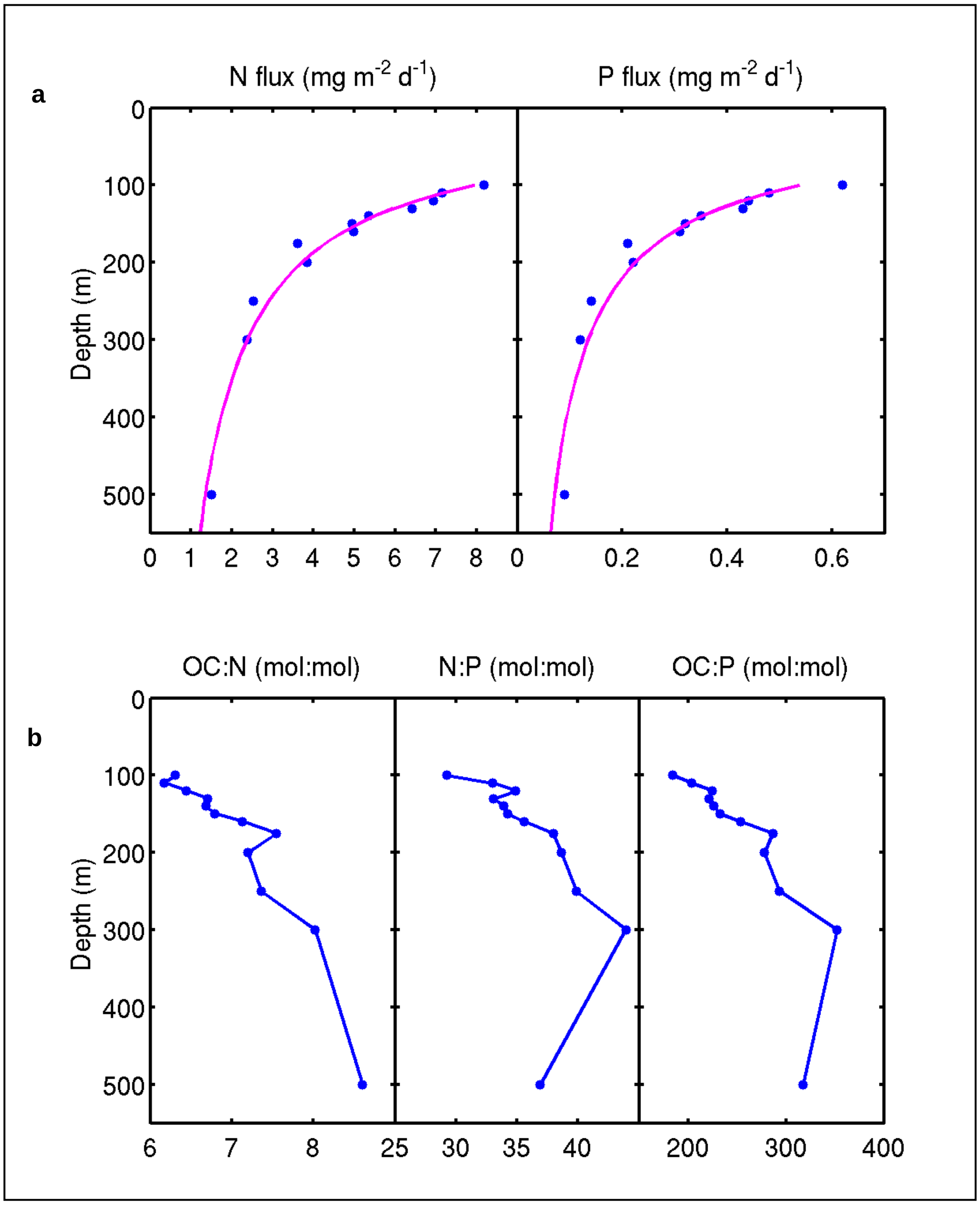

Supplementary Figure 2. Elemental fluxes and stoichiometries. a Flux profiles of nitrogen (N) and phosphorus (P) for the particulate matter collected in the free-drifting sediment trap experiment. The magenta curves are the best fits to a log-log transformed (Supplementary Data Fig. 1) normalized power function of the form: $F_z = F_{100}\ (z/100)^b$ where F_z is the flux at depth z(m), F_{100} is the flux at 100 m and b is the coefficient of flux attenuation (ref. 11). The best fit parameters are: N Flux, F_{100} = 7.94 mg N $m^{-2}\ d^{-1}$, b = –1.10, r^2 = 0.95; P Flux, F_{100} = 0.54 mg $m^{-2}\ d^{-1}$, b = –1.26, r^2 = 0.90. **b** Depth profiles of the OC:N, N:P and OC:P molar ratios for particulate matter collected in the free-drifting sediment trap experiment.

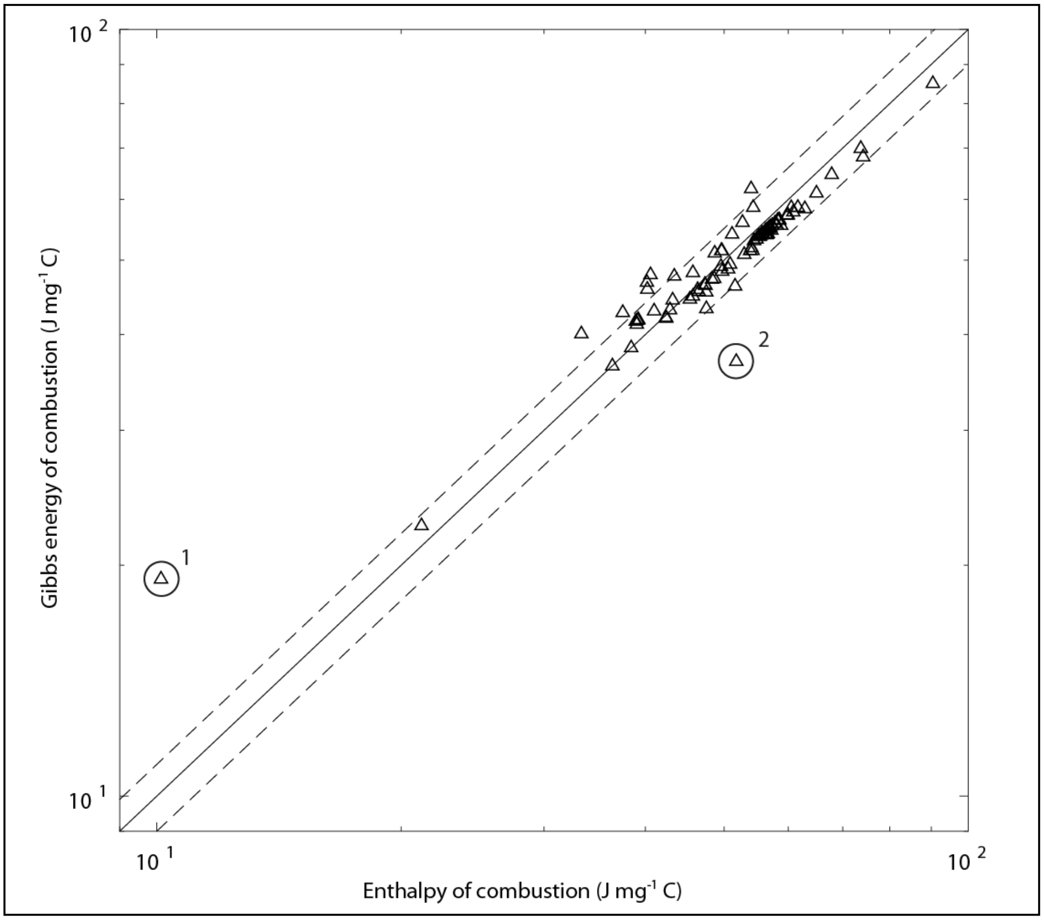

Supplementary Figure 3. Comparison of enthalpies of combustion and Gibbs energies of combustion for a variety of organic compounds based on literature values (https://www.chemeo.com). The 91 organic compounds include: methane, ethane, propane, butane, pentane, hexane, heptanes, octane, nonane, decane, undecane, dodecane, 2-methyl propane, 2-methyl butane, 2-methyl pentane, 2-methyl hexane, 2-methyl heptanes, 2,2-dimethyl propane, cyclopropane, cycopentane, cyclohexane, ethane, propene, but-1-ene, trans-but-2-ene, cisbut-2-ene, buta-1,2-diene, buta-1,3-diene, phenylethene, ethyne, propyne, benzene, methylbenzene, ethylbenzene, propylbenzene, 1,2-dimethylbenzene, 1,3-dimethylbenzene, 1,4-dimethylbenzene, ethenylbenzene, methyl amine, dimethyl amine, trimethyl amine, 1amino butane, methanol, ethanol, propan-1-ol, propan-2-ol, butan-1-ol, pentan-1-ol, hexan-1-ol, heptan-1-ol, octan-1-ol, ethan-1,2-diol, cyclohexanol, methoxymethane, ethoxyethane, methanol, ethanol, propanal, butanal, acetone, butanone, methanoic acid, ethanoic acid, propionic acid, glutamic acid, glycine, serine, CO_2, urea, cyclooctratetraene, nitrobenzene, phenol, pyridine, oxalic acid, benzoic acid, valine, phenylalanine, threonine, leucine, isoleucine, lysine, proline, tyrosine, methionine, cysteine, aspartic acid, asparagine, glucose, sucrose, fructose and lactose. The solid line is the 1:1 relationship and the dashed lines represent ±10% of the 1:1 line. The two circled values are (1) cyclohexanol and (2) oxalic acid.

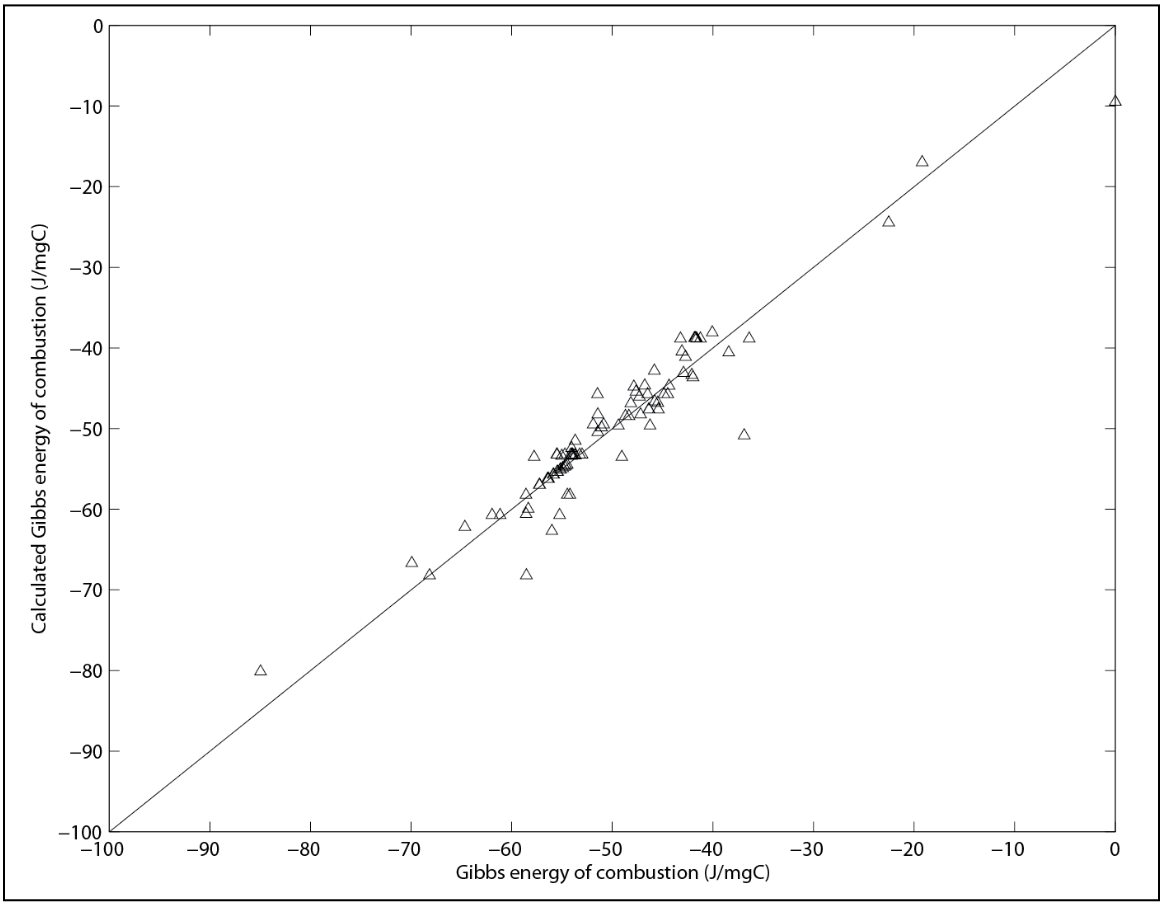

Supplementary Figure 4. Gibbs energy of combustion. A comparison of the literature values for Gibbs energy of combustion and the calculated Gibbs energy of combustion based on the C, H, N, O, S elemental composition for the 91 organic compounds in Supplementary Data Fig. 1. The solid line is a 1:1 fit to the data.
